# Needs to Create Healthy Living Environments—A Two-Stage Delphi Survey in Europe to Identify Facilitating Factors and Barriers in Municipal Health Promotion

**DOI:** 10.3390/ijerph19095084

**Published:** 2022-04-21

**Authors:** Eike Quilling, Maja Kuchler, Patricia Tollmann, Anke Osterhoff, Janna Leimann

**Affiliations:** Department of Applied Health Sciences, Hochschule für Gesundheit—University of Applied Sciences, 44801 Bochum, Germany; maja.kuchler@hs-gesundheit.de (M.K.); patricia.tollmann@hs-gesundheit.de (P.T.); anke.osterhoff@hs-gesundheit.de (A.O.); janna.leimann@hs-gesundheit.de (J.L.)

**Keywords:** Delphi survey, health promotion, inequalities, municipalities, communities, participation, collaboration, health equity

## Abstract

(1) In the field of health promotion, municipalities offer opportunities to reduce SES-based health inequalities by addressing vulnerable communities. This research project aims to identify facilitating and inhibiting factors for the creation of healthy living environments. (2) After preliminary literature and qualitative research work, an online-based Delphi survey was conducted (December 2020–March 2021). This included the rating and commentating of 22 theses at two times, whereby the results of the first round of rating were visible to the participants the second time. (3) Twelve experts from seven European countries participated in the Delphi survey across both rounds (1st round: *n* = 37; 12 countries). The consensus was particularly clear with regard to providing resources, which, in turn, are especially necessary for involving target groups in health promotion. (4) The results illustrate the relevance of further cross-national exchange. Certain aspects however, such as the HiAP approach or strategies to reach disadvantaged groups, are still challenging in practice. In order to develop concrete recommendations, the theses need to be further operationalised. The Delphi method offers a suitable possibility to map international expertise in this field and with a focus on health equity.

## 1. Introduction

Health continues to be unequally distributed and the social gradient plays a major role in determining peoples’ health status [1,2]. The COVID-19 pandemic, in particular, showed very clearly that both health status and the ability to access health care depend on socio-economic status: People with lower income and lower educational levels are more likely to suffer from COVID-19. For example, they are significantly more likely to work in systemically relevant occupations, where there is less opportunity to work from home or avoid contact [3]. Additionally, health services are significantly underrepresented in disadvantaged neighbourhoods [1]. To avoid widening this gap, it is particularly important to create frameworks and interventions that focus on disadvantaged and vulnerable groups to improve their health equity. Municipalities offer the chance to remedy this situation and to reach all people in their living environment with health-promoting measures [4,5]. As an umbrella setting, the municipality offers ideal conditions for health promotion, especially with regard to reaching vulnerable groups: on one hand, it is home to many different groups of people who can be reached by setting-based actions. On the other hand, the inclusion of diverse, often specific settings makes it easier to reach marginalised target groups [6,7]. In a scoping review, Quilling et al. [8] present the state of evidence on municipal health promotion, including examples of successful health promotion programs at the municipal level. One of the key findings is that evidence-based theoretical models and approaches provide guidance but do not guarantee the success of municipal health promotion. Reviewing international scientific reports in the field of municipal health promotion, it becomes clear that different strategies are available. These are, e.g., the creation of networks [9,10,11,12], capacity building [10,13,14], structure building [11,15], participation [16,17], reaching the population [14], joint decision-making [12,18], local planning and implementation, and the creation of resources [18].

Despite the aforementioned advantages that the structure of municipalities brings, there are often also challenges to overcome in municipal health promotion: the diversity and quantity of residents and actors in and around a municipality makes communication difficult [18]. Often, numerous similar interventions coexist in a municipality and, thus, make the field intransparent and complex, especially for residents. Such double structures with different actors also make it difficult to coordinate an integrated approach [19]. The goal of an overall municipal strategy that coordinates the existing support services and health-promoting offers across all phases of life can only be achieved if all local actors work together, network across activities, and create preventive measures that effectively help residents through the division of labour and joint planning [20].

Municipal approaches are also gaining importance in the face of demographic change and the associated increase in chronic diseases and multimorbidity in the population. They represent a valuable opportunity to influence the health of an aging population. Röding, Walter, and Dreier investigated the long-term effects of integrated municipal health promotion strategies on mortality associated with diabetes mellitus and were able to present initial evidence of the effectiveness of integrated municipal health promotion strategies in Germany [21]. Coordinated linking of different sectors within the municipality is required in order to support a multimorbid, aging population, and to improve their participation and quality of life. This is not just a matter for health care professionals alone, but also and more particularly for the social sector.

Health should be understood as a task for society as a whole: The creation of healthy living environments is not exclusively in the charge of the health sector. Following the Health in All Policies approach (HiAP), other sectors are also responsible and should be involved in all policy decisions that have an impact on the health of the population [2,22]. Apart from policy decisions, health determinants can be changed through joint activities of strategic partners from different sectors and at different levels of government. It is observable that the complexity of this interplay of actors from different sectors, policy areas, and professions often leads to specific challenges in the process of municipal health promotion. Research results indicate that these challenges are often very similar [18,23]. Thus, there seem to be phases in the process that are more difficult to implement than others, examples include the participatory planning phase and evaluation.

Although there are several strategies to facilitate the successful implementation of municipal health promotion, there is a lack of practical recommendations to help implementing these strategies and recommendations to address the challenges identified [18,23].

Linking the present research project to a part of the European project “Joint Action Health Equity Europe (JAHEE)” enabled the Hochschule für Gesundheit (University of Applied Sciences), Bochum, Germany to address the issue of health equity in municipal health promotion across countries: JAHEE provided an important opportunity for member states to work together to address health inequalities and achieve more equity in health outcomes across all social groups, in all participating countries, and in Europe as a whole [24].

The accompanied part of JAHEE focused on assisting member states in identifying national strategies and policies and in developing a guide for decision-makers and stakeholders for creating healthy living environments. The present project carried out at the University of Applied Sciences aimed to complement this part of JAHEE with concrete recommendations for creating health equity at the local, municipal level. The existing alliance of European member states in JAHEE offered ideal opportunities to access and make use of the expertise of various international stakeholders.

The aim of this article is to present selected results of an international Delphi survey conducted in this context. This serves to substantiate the results with regard to the overarching research questions: which facilitating and inhibiting factors can be identified in the implementation of health-promoting environments in municipalities (across countries)? Which concrete recommendations for action can be derived that support actors and decision-makers in setting the course for the promotion of healthy living environments?

## 2. Materials and Methods

The Delphi method represents a possibility to collect the targeted expertise of both scientists and practitioners. This method is commonly used in technology and politics as a forecasting instrument and is often used in the health sciences as a consensus procedure [25,26].

In this procedure, experts are questioned on various topics in a multi-stage survey process. Respondents are provided with information on the entire group’s response behaviour in each round of questioning. This gives them the opportunity to reflect on their own opinion in the process and possibly change or consciously retain it [27]. Thus, the Delphi method aims to establish dissent or consensus on dissent among the responding experts [28].

### 2.1. The Delphi Survey as a Participatory Method for Approaching the Research Questions

The core of the Delphi survey presented here was to evaluate and comment on theses around the topic of “creating healthy living environments in the municipal context”. The aim was to gain insights into how the different countries involved deal with challenges, such as the inclusion of marginalized groups. Participants were anticipated to benefit from taking part in the associated discussions and learning from each other’s approaches to tackling health inequalities. The Delphi survey consisted of two rounds conducted between December 2020 and March 2021. In preparation for the Delphi survey and the development of the theses, a rapid review on the design of healthy living environments, as well as guideline-based expert interviews and an expert workshop with the JAHEE cooperation partners were conducted. This rapid review served to identify initial thematic focal points, which were further deepened and, if necessary, expanded during the interviews. Based on this, the researchers generated initial theses for the Delphi survey which were jointly reviewed and further developed in the expert workshop with regard to content and comprehensibility.

Thesis selection aimed at covering all relevant topics and avoiding redundancy in the Delphi survey.

A further, final step was another meeting with the experts after completion of the Delphi survey: experts were presented with the final results and initial ideas for concrete recommendations generated on the basis of the results of the Delphi survey and were given the opportunity to comment on them and, if necessary, make further remarks.

### 2.2. Thematic Focal Points in the Development of Theses for the Delphi Survey

In the preliminary work for the Delphi survey, 22 theses were developed and clustered into five thematic areas. Each of the areas contained between three and six theses (Table 1):

- Networks;
- Analysis and strategy development;
- Working principles for health promotion;
- Evaluation;
- General issues.

**Table 1:** Overview of the theses included in the Delphi survey.

	Nr.	Thesis
**Networks**	1	Without additional resources it is impossible to build networks.
	2	Clear criteria must be defined to identify suitable network partners.
	3	Networks in municipal health promotion should remain open in order to react flexibly to changes and developments (in the process).
	4	A common vision (considering the different perspectives, knowledge, and experiences of the partners) is necessary for planning and implementing actions.
	5	Having the same contact persons (as permanent correspondent) and consistent processes for health promotion in a municipality is essential for project work.
**Analysis and strategy development**	6	Actions must be based on scientific evidence for successful municipal health promotion.
	7	Actions can be based entirely on individual experience of the practitioner.
	8	An overall strategy at national level is necessary for the realization of the health promotion process in a municipality.
	9	An overall strategy at national level has to consist of several individual components that can be implemented needs-based and in parts.
	10	Needs analyses within the municipality are always indispensable.
	11	It is at least as important to find out what is feasible as what is needed in the municipality.
**Working principles for health promotion**	12	If there is competition within networks of municipal health promotion, it is required to communicate this openly.
	13	Without the involvement of the target group(s), actions cannot be implemented successfully.
	14	The organisation of participation processes requires time, financial and human resources.
	15	There is a lack of awareness that municipal health promotion should aim at reaching vulnerable population groups.
	16	Without a concrete strategy, vulnerable population groups cannot be identified, reached, and involved in the process.
**Evaluation**	17	A successful evaluation can only be accomplished if concrete objectives are set at the beginning.
	18	Decision-makers and funders need knowledge about measurable health outcomes in order to have realistic expectations regarding possible effects (which are collected in the evaluation).
	19	A continuous process evaluation of activities is required to be able to ensure quality control.
**General issues**	20	The implementation of municipal health promotion requires a centralised professional overall coordination.
	21	In order to implement an overall municipal strategy for health promotion, actors from all policy areas must feel responsible.
	22	The successful planning and implementation of municipal strategies can only be accomplished with professionally qualified and experienced personnel.

### 2.3. Recruitment and Selection of Suitable Participants

The recruitment of participants for the Delphi survey was carried out by E-Mail. Primary addressees were JAHEE project partners who all are experienced professionals in municipal health promotion in their respective European countries (13 total). In this way, many experts in the different European contexts could be reached and activated. The addressed experts were mainly decision-makers with coordinating tasks but also professionals from the field of practical implementation of health promotion projects and science. An accompanying factsheet, with information on the aim and modalities of the Delphi survey, was sent out with the survey. In addition, the experts were asked to invite other colleagues to participate in the Delphi survey in the sense of a snowball principle. For this purpose, an information text to be forwarded was also provided in the mailings.

### 2.4. Design of the Online Questionnaire to Collect Relevant Results in the Delphi Survey

To collect the data, the experts completed an online questionnaire at both times of the Delphi survey. The survey was implemented with the survey tool LimeSurvey 3.27.6 due to its particular suitability in terms of data security, feasibility in English, and display options.

**Figure 1:**
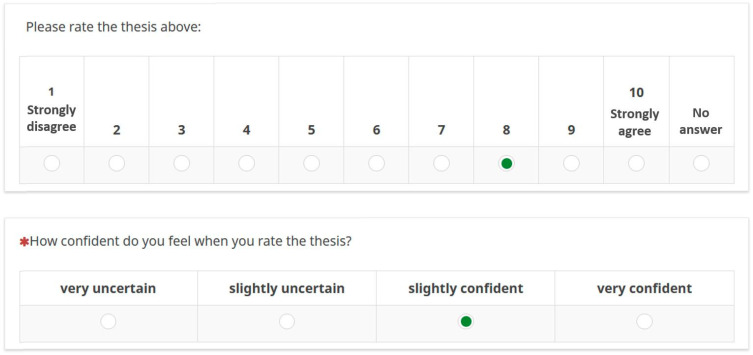
Ten-point rating scale for agreement with the thesis and four-point rating scale for indication of confidence in rating. (* = Question was mandatory if the thesis was rated with a value between 1 and 10).

Following the recommendations of Schulz and Renn [29], a 10-point rating scale for each thesis (1 = strongly disagree to 10 = strongly agree) was used. In addition, the item “No answer” was used as an alternative category in order not to force responses. If the participants rated the thesis with a score between 1 and 10, their confidence while rating was also queried on a 4-point scale (“very uncertain” to “very confident”) in order to identify potentially uncertain opinions and to be able to interpret the results in further detail for later discussion (Figure 1). An ordinal level of measurement was chosen because methodologically no scoring of the rating with the confidence check was planned.

In addition to the quantitative part of the questionnaire, there was an optional opportunity to comment on each thesis and to add recommendations and further aspects at the end to collect supplementary qualitative data.

Participants were asked to generate their own identification codes for pseudonymization purposes. This made it possible to compare responses from both rounds of rating within participants. Rating in the first round was estimated to take about 15 min. Participants were allowed to interrupt answering and to continue to a later time. For the second round, it was pointed out that a minimally larger amount of time might be necessary due to the more complex task of interpreting and reviewing presented data.

### 2.5. Data Analysis Procedure

**Figure 2:**
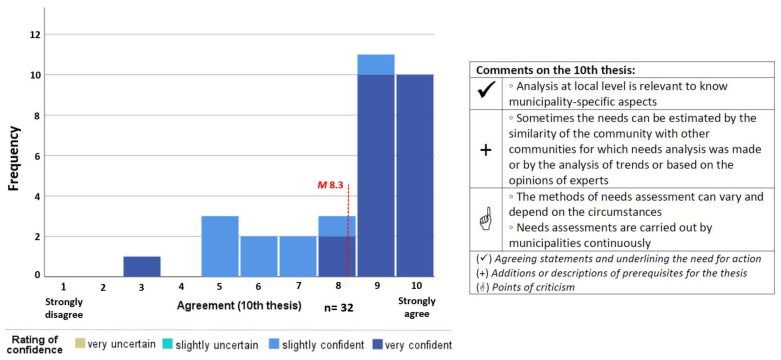
Example of how the results from the first round of interviews were presented in the second round of the Delphi survey.

After the first round of data collection, the quantitative parts of the questionnaire were analysed descriptively with SPSS (IBM SPSS Statistics 27) and the qualitative parts were analysed manually regarding content. Both sets of data were summarised for each thesis in order to make the results visually presentable for the second round of data collection: in order to visualise both the frequency of the individual evaluations and the results of the confidence test in one diagram, stacked bar charts were used. In addition, the mean value of the ratings for the respective thesis was marked (Figure 2). Qualitative data were summarised in a table and divided into the following three main areas:

- Agreeing statements;
- Additions;
- Points of criticism.

This type of representation was created for each individual thesis of the Delphi survey and embedded in LimeSurvey so that the evaluation behaviour from the first round was transparent in the second survey round.

After completion of the second survey round, the researchers again evaluated the results quantitatively and qualitatively. In the following, the changes in thesis-rating (based on changes in mean values), the results of the confidence check, and the supplementary qualitative answers or comments given by the participants were prepared.

As quantitative items made up the majority of the survey, a statistical determination of a consensus was planned. To determine the extent of consensus building, the standard deviations of the first and second round results were compared. The Delphi survey process was expected to lead to an increase in consensus and, thus, a decrease in dispersion [30]. In addition, rating mean values provide information on whether participants tended to agree or disagree with the presented theses.

## 3. Results

In the following, the key results of the Delphi study are shown to evaluate the theses on inhibiting and facilitating factors, which were identified and developed in the preliminary work. After a brief overview of the characteristics of the participating experts, the presentation of key results will follow.

### 3.1. Participation in the Study

**Figure 3:**
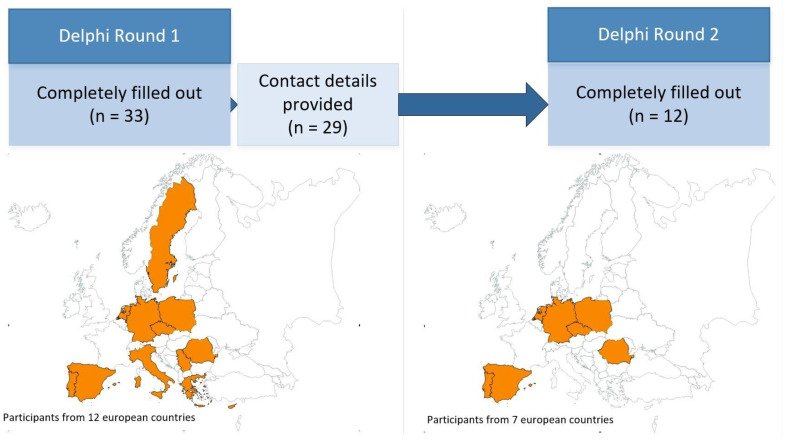
Presentation of the number of participants in the two Delphi survey rounds from the European countries involved.

In total, 37 experts participated in the first round of the Delphi survey, 33 of whom completed the survey in full. Overall, 29 of the remaining 33 participants left their contact information for participation in the second round of the survey. Out of these 29 participants, 15 took part in the second round, but only 12 completed the entire survey. The 12 participants who completed both rounds each consisted of four from Southern, Central, and Eastern European countries, respectively (Spain, Portugal, Germany, Netherlands, Poland, Czech Republic, and Romania) (Figure 3). Seven experts identified themselves as working in the field of public health, two in the field of health promotion, and one each in the fields of environmental policy, urban planning, and health care in general.

### 3.2. Presentation of Selected Results of the Delphi Survey

Key results include information on consensus-building in general, mean values and standard deviations of the second survey round (*n* = 12), as well as comments and remarks that explain the controversial opinions in the two survey rounds and further enhance the theses. The presentation thereof corresponds to the five thematic areas identified earlier. Finally, the results are described with regard to the reported confidence during rating.

### 3.3. Consensus Building

Consensus was determined in the present study if standard deviations in the second round were less than or equal to 1.4. This limit seemed appropriate to the researchers by comparing it with corresponding literature [29,31]. With a standard deviation of ≤1.4, a uniform opinion in the sense of a consensus was assumed in this Delphi survey. Standard deviations >1.4 tend to indicate a wide range of opinions and indicate that there is no consensus among the experts.

In terms of the development of the standard deviation in rating the theses from the first to the second round, a reduction in the dispersion in the sense of a decrease in the standard deviation occurred in 19 of the 22 theses. At the end of the process, 10 theses were found with high agreement (rating ≥ 7) and low standard deviation (consensus), 9 theses with high agreement (rating ≥ 7), and higher standard deviation (smaller consensus) and only 3 theses with medium agreement (rating 4–6) and high standard deviation (rather disagreement):

- Theses with high agreement and low standard deviation:
  → Theses 3, 4, 10, 11, 13, 14, 16, 18, 21, and 22.
- Theses with high agreement and higher standard deviation:
  → Theses 1, 2, 5, 6, 8, 12, 17, 19, and 20.
- Theses with medium agreement and high standard deviation:
  → Theses 7, 9, and 15.

### 3.4. Topic 1: Networks

With regard to networking, the participants agreed that networks in municipal health promotion should remain open in order to be able to react flexibly to changes and developments in the process (Thesis (T) 3: *M* 9.17; *SD* 0.6). In addition, most experts agreed that a shared vision (taking into account the different perspectives, knowledge, and experiences of partners) is necessary for planning and implementing actions (T4: *M* 8.33; *SD* 0.8). The need for additional resources to build networks (T1: *M* 7.08; *SD* 2.2) and the definition of clear criteria for identifying network partners (T2: *M* 7.75; *SD* 1.7) were rated more controversially. Resources, according to stakeholder comments, were helpful but not a requirement. The experts reasoned that groups of people who stand to benefit from networks would participate in them voluntarily. Financial resources were seen as only necessary for the implementation of activities. They pointed out that in addition to motivation and willingness to participate, the selection criteria should only be developed in the process with the partners and should be critically considered under the aspect of open network design. The participants disagreed about the essentiality of permanent contact persons and constant processes for health promotion in cities for project work, but largely agreed with the thesis (T5: *M* 7.91; *SD* 1.9). High staff turnover in projects was noted critically. It is all the more relevant to update contact lists and to extend responsibility and accessibility from one person to a team.

### 3.5. Topic 2: Analysis and Strategy Development

In the field of analysis and strategy development, there was a high level of consensus regarding the indispensability of needs assessments (T10: *M* 8.92; *SD* 1) and testing of feasibility (T11: *M* 8.83; *SD* 0.8). There was agreement, but less uniformity, with regard to the need for actions to be based on scientific evidence (T6: *M* 7.42; *SD* 2). It was critically noted that the concept of evidence should be broad, that the knowledge of stakeholders and participants should also be included, and that space for participatory methods was necessary. Furthermore, it was noted that scientific evidence (in municipal health promotion) is not always available and that health promotion is a complex challenge that cannot always be addressed with only science-based interventions. Sometimes it can be useful to test approaches with little existing evidence in order to generate (more) evidence. The participants also hold controversial views regarding the need for overall strategies at national level for the implementation of municipal health promotion (T8: *M* 7.5; *SD* 1.8). They agreed even less with the thesis that such an overall strategy should consist of various individual components that could then be implemented as needed (T9: *M* 6.83; *SD* 2.1). The fact that an overall strategy would be helpful, but not necessary, was emphasised several times in both rounds of the Delphi survey. According to the more critical participants, possible individual strategy components should only be formulated after the formulation of the overall strategy is complete. The thesis that actions can be based solely on one practitioner’s experience met with the lowest level of agreement (T7: *M* 4; *SD* 3). In this context, stakeholders noted that individual experiences could be a starting point for action, but that these should always be contextualised and further developed and implemented with the involvement of other stakeholders and in line with scientific evidence and best practice. The complexity of health issues requires interdisciplinary, cross-sectoral collaboration, including residents and policy-makers.

### 3.6. Topic 3: Working Principles for Health Promotion

Regarding the working principles for health promotion, there was a high level of agreement on the need for time, financial and human resources (T14: *M* 9.5; *SD* 0.9), with the latter being particularly emphasised in the comments. Agreement levels were somewhat lower, but still relatively high and consistent, for the thesis that target groups must be involved so that actions can be successfully implemented (T13: *M* 8.83; *SD* 1.4). However, this is only viewed as possible if the corresponding resources are available, but then leads to a high acceptance of the projects. Stakeholders also fairly agreed that concrete strategies are needed to identify, reach, and engage vulnerable groups in the process (T16: *M* 8.73; *SD* 1.1). Important resources, as part of the strategy, mentioned here were professionals, such as social workers, who have privileged access and acceptance in these groups. The participants’ opinion was diverged a little more with regard to the fact that there is competition in municipal health promotion and that it is necessary to communicate this openly (T12: *M* 8.4; *SD* 1.7). Evaluation of participants‘ responses made it clear that for many it was unimaginable that there was such a thing as competition within networks of municipal health promotion in the field. The thesis that there is a lack of awareness that municipal health promotion should target vulnerable populations was largely criticised by the participants (T15: *M* 6.83; *SD* 1.9). Stakeholders noted that while it is sometimes forgotten that some groups need a special focus, the bigger challenge is reaching vulnerable groups because existing strategies are not feasible. They also reported a lack of resources and prestige as barriers to effective targeting of vulnerable groups.

### 3.7. Topic 4: Evaluation

In the area of evaluation, the theses put forward were rated fairly similar. Most participants strongly agreed that decision-makers and funders need knowledge of measurable health outcomes in order to have realistic expectations of the potential impact (captured in the evaluation) (T18: *M* 8.0; *SD* 1.3), that concrete objectives need to be defined at the beginning for a successful evaluation (T17: *M* 8.33; *SD* 1.6), and that continuous process evaluation of activities is necessary to be able to ensure quality control (T19: *M* 8.33; *SD* 1.8). The measurability and change of the environmental conditions and the rapid developments, especially in the neighbourhoods of the vulnerable groups, were mentioned as challenges for all these aspects.

### 3.8. Topic 5: General Aspects

There was particularly clear agreement with the thesis that actors from all policy areas must feel responsible in order to implement an overall strategy for municipal health promotion (T21: *M* 9.08; *SD* 0.8). Furthermore, many stakeholders agreed that qualified and experienced personnel are needed for successful planning and implementation (T22: *M* 8.33; *SD* 1.4). More controversial was the thesis that this also requires centralised professional overall coordination (T20: *M* 8.08; *SD* 1.8). Some felt that this role facilitates the implementation of health promotion, while others see centralisation as an obstacle and a step away from action and responsibility at the local level.

### 3.9. Confidence while Rating the Theses

**Figure 4:**
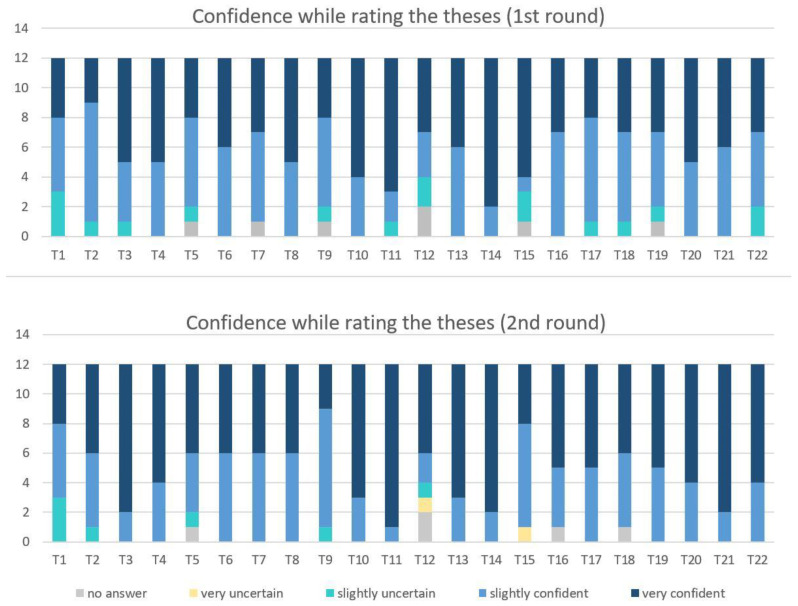
Confidence while rating the theses in the first and second round.

Figure 4 shows in two diagrams the confidence of the participants who took part in the first and second Delphi survey round while rating the theses. Comparing the two, the general tendency is that the confidence in the evaluation increased from the first to the second round. For 16 theses, the experts’ confidence was higher in the second round. For three theses, the feeling of confidence remained the same (T1, T6, T14), although the confidence was already high (T6) or very high (T14). For the other three theses (T8, T9, T15) the results show uncertainties. These were theses that were evaluated rather controversially in the first round (SD ≥ 2.5). For a total of 14 theses, the participating experts were only slightly or very confident in their answers in the second round. For the remaining eight theses, there were isolated uncertainties and some abstentions in the evaluation. Confidence was particularly high (≥75% of the answers “very confident”) at the end of the second round for eight theses (T3, T10, T11, T13, T14, T20, T21, T22).

## 4. Discussion

Based on the results presented previously, the method of the present Delphi survey will be discussed below. Beneficial aspects in the methodological approach as well as obstacles are highlighted.

### 4.1. Discussion of the Method

The Delphi method was found to be a suitable, albeit complex, method for the research project. Extensive preliminary work (literature research, interviews, and expert workshop), which is not necessarily intended in Delphi methods, proved to be time-consuming but was very conducive to the resulting quality of the study. Thus, relevant topics could be thoroughly researched and theses developed, which were then able to be specified through the Delphi process.

The connection to the EU project JAHEE enabled easy and low-threshold access to participating experts from the health sector, which would otherwise have been rather challenging in the 2020 COVID-19 pandemic situation. This Delphi survey nevertheless showed a high attrition rate. According to Mullen [32], there is no consensus on what is an acceptable attrition rate in a Delphi process. Additionally, according to Nasa, Jain, and Juneja [33], there is no standard size for the participant group—but typically it ranges from 10 to 100. The sharp decrease in participants and participating countries from the first to the second round is certainly due to the fact that the pandemic situation intensified again in the winter of 2020/2021 and the participants on the ground were involved in pandemic management and containment as community health workers. Moreover, the second survey coincided with the final phase of the EU project JAHEE and the cooperation partners needed time and resources to complete their own projects. Lack of time is also highlighted by Sherwood, Deery, and Jago [34] in their research as the main reason for non-response. Gargon et al. [35] also associate lower response rates with a high item count. However, the researchers would rate the number of items as appropriate in this study. In contrast to a group Delphi procedure in presence, the online version of the Delphi survey allowed for unbiased rating. The expert workshop for participatory discussion and further development of the first version of the theses for the Delphi survey enabled a targeted specification of the theses and produced a clear number of theses. It can be assumed that this led to good comprehensibility in the subsequent consensus-finding and thus resulted in a quick and predominantly clear formation of opinions.

The possibility of making comments in both rounds and visualising them in the second round of the survey proved to be a practicable and very advantageous solution for further developing and specifying the theses in the evaluation. This added value of qualitative elements is increasingly recognised in Delphi studies [28]. According to the participants’ feedback, the visualisation of the results seemed to have been chosen appropriately, seeing as only a few questions regarding the way of presentation were reported.

The small number of participants and the high attrition rate represent limitations of the study. They may have had an impact on the final results [36] and must be considered when interpreting them. Nevertheless, expertise and experience from seven different countries flowed into the results and it was possible to draw from extensive qualitative responses.

The inclusion of only European countries or experts means that the transferability of results is limited to other European countries or countries with similar municipal structures and health care systems. The present results do not have general validity, but they led to a good understanding among the surveyed experts.

The aim of this study was not only to reach a consensus in the statistical sense, but also to explore the topic in greater depth and to gain insight into the experts’ views, which was made possible very well not least by the qualitative elements. 

### 4.2. Discussion of the Results

After discussing the methodological approach, the results concerning the facilitating and inhibiting factors are discussed below. Furthermore, country-specific similarities, differences, and further research directions will be highlighted.

#### 4.2.1. Similar Challenges Exist across Countries

In the context of this Delphi survey, there was a consensus tendency with high confidence in the evaluation for most of the theses presented, which illustrates the cross-national similarity of experiences with the challenges and needs for development in municipal health promotion. The consensus was particularly high with regard to the conducive factors of openness in the design of networks and the inclusion or responsibility of all political sectors in the design of healthy living environments. The relevance of the latter has been discussed and demanded in the efforts for the HiAP-approach in the professional world for years [2,22] and seems to be seen as similarly relevant in all participating countries. The high demand for resources for the implementation of actions was uniformly highlighted as a challenging factor. Reaching marginalised populations is also experienced as challenging and at the same time perceived as particularly relevant in all participating countries. The fact that, as the results made clear, evaluating measures in disadvantaged areas is difficult in the face of their rapid change makes it all the more challenging to test and evaluate measures and strategies there. The similarity of experiences and ratings underlines what was also experienced and described by our participants in the expert workshops: the benefits of exchanging views and learning from different experiences in other European countries.

#### 4.2.2. Experience and Country-Specific Perspectives

The results also show the heterogeneity and diversity of municipal health promotion in the countries depending on the given framework conditions and structures in the country of impact or also on the participants’ own profession. For example, in the discussion about centralising the overall coordination of health promotion in the municipality, it became apparent that the participants had differing interpretations of the task of a coordinating person and have partly also had different experiences with centralised processes in their countries. It was striking that the experts were particularly self-confident in their respective different opinions. This shows that scientists have to be sensitive to different structures and experiences and that solutions cannot necessarily be applied across the board. Similarly, some participants did not realise until the end of the Delphi process that there is or could be competition in the field of health promotion. This seems to be experienced differently due to different funding conditions in the participating countries and more or less competing providers in the respective system.

#### 4.2.3. Expertise from the Field Offers Opportunities for Sharpening the Theses That Have Been Formulated in the Delphi Process

Overall, the evaluation and commentary of the work reveals the experts’ views of their practical experience which they bring with them (mostly as decision-makers) from the implementation, monitoring, and coordination of various projects and contexts. This practical relevance is reflected, for example, in the opinion that additional resources for the formation of networks are helpful but not a prerequisite, as the right partners can often contribute the most. However, the assessment is different with regard to the resource requirements for the implementation of measures, which the practitioners uniformly rate highly. It also becomes evident from the evaluation of the contact persons and the uniform process design in the municipalities that the participants incorporate experiences and propose appropriate solutions, such as the division of tasks in the team, to counteract problems that can arise, for example, due to staff changes. The controversial opinions and the discussion about the evidence-based characteristics of interventions show that the participants experience a great challenge in practice. As often described in professional discourse [18,37,38,39], from their point of view there seems to be a great need to improve the evidence base for creating health-promoting environments. The counter-thesis that actions can also be based on purely practical experience, on the other hand, was also weakened. Critically, this thesis was somewhat misleadingly formulated, so that participants explicitly pointed out that actions should not be based on the experiences of individual practitioners. However, even “best” or “promising practice”, which is often, though not entirely uncritically, regarded as the best available evidence in municipal health promotion [40], was cautiously described here as a possible basis for action under the aspects of contextualisation and transferability. With regard to the evaluation of projects in municipality structures and neighbourhoods, the complexity and also the rapidly changing framework conditions were explicitly pointed out here as well [18,37,38,39]. Although evaluation is seen as relevant to practice, it is also perceived as very challenging.

### 4.3. Significance of the Study Concerning Chronic Diseases

Successful cross-sector collaboration offers the opportunity to address increasing (multi-)morbidity by aligning services across age groups and life stages. The present study provides guidance on facilitating factors and barriers in municipal health promotion, the core of which is the creation of an integrated overall strategy, to structurally address the determinants of health. This study also provides insight into the feasibility of a method that can be applied in the context of collaboration to address and bring together multifaceted opinions through a participatory process, also cross-national. This is particularly relevant for the cooperation of the different professions and sectors in order to jointly prevent chronic diseases, as these represent an internationally overarching challenge [41].

### 4.4. Further Need for Action and Research

In the further course, the findings from the Delphi survey will need to be further developed into concrete recommendations for action. For transferability into the structures of policy and practice, these must be further operationalised. For this purpose, surveying larger samples could provide deeper insights. Furthermore, it would be interesting to investigate to what extent the present results remain valid beyond the European context. It became clear that there is a need for further exchange of views and networking across national borders.

## 5. Conclusions

Based on this international Delphi survey, it was possible to identify various promoting and inhibiting factors in the field of municipal health promotion with a focus on health equity across countries. Extensive preliminary work and the additional collection of qualitative data in the Delphi survey led to a refinement of the theses and increased their validity. As key findings of the Delphi process it became evident that:

- Additional resources are needed for the participation of target groups in all stages of the project process and to identify and adequately reach marginalized groups;
- Stakeholders from all policy areas should be involved in the implementation of an overall municipal strategy;
- Open networks and having a common vision are highly relevant in municipal health promotion;
- More evidence is needed to implement health promotion interventions in municipal contexts.

The innovative part of the recommendations is the participative process with international experts that enabled a cross-national consensus and compilation which were further developed as recommendations for action.

## Data Availability

Not applicable. The technical report is not published yet.
